# DHX34 as a promising biomarker for prognosis, immunotherapy and chemotherapy in Pan-Cancer: A Comprehensive Analysis and Experimental Validation

**DOI:** 10.7150/jca.102230

**Published:** 2024-10-28

**Authors:** Nanbin Liu, Qian Wang, Pengpeng Zhu, Gaixia He, Zeyu Li, Ting Chen, Jianing Yuan, Ting La, Hongwei Tian, Zongfang Li

**Affiliations:** 1 National and Local Joint Engineering Research Cente of Biodiagnosis and Biotherapy, The Second Affiliated Hospital of Xi'an Jiaotong University, Xi'an, China.; 2 Shaanxi Provincial Clinical Research Center for Hepatic & Splenic Diseases, Xi'an, China.; 3 Department of Geriatric General Surgery, The Second Affiliated Hospital of Xi'an Jiaotong University, Xi'an, China.; 4 Tumor and Immunology center of Precision Medicine Institute, Xi'an Jiaotong University, Xi'an, China.

**Keywords:** Pan-Cancer, DHX34, Prognosis, Chemotherapy, Immunotherapy

## Abstract

**Background:** As a member of the DExD/H-box RNA helicase family, DHX34 has demonstrated a significant correlation with the development of multiple disorders. Nevertheless, a comprehensive investigation between DHX34 and pan-cancer remains unexplored.

**Methods:** We analyzed the value of DHX34 in pan-cancer based on some databases, such as The Cancer Genome Atlas (TCGA), Gene Expression Omnibus (GEO), and The Human Protein Atlas (HPA) by use the R language as well as some online analysis tools, including STRING, TISIDB, TISCH2. And based on our samples we performed Western blot (WB), qPCR and immunohistochemical staining (IHC) experiments.

**Results:** DHX34 was highly expressed in most tumors, including Liver Hepatocellular Carcinoma (LIHC), compared to corresponding normal tissues. Among cervical cancers, DHX34 mutation frequency was the highest. Intriguingly, a positive correlation was observed between DHX34 expression and Mutational Burden (TMB) across 12 tumor types, and Microsatellite Instability (MSI) across 10 tumor types. Remarkably, DHX34 exhibited a favorable diagnostic value in a multitude of tumors. High expression of DHX34 is associated with poor prognosis in tumors such as adrenocortical carcinoma (ACC), renal papillary cell carcinoma (KIRP), low-grade glioma (LGG), and LIHC. Correlation analysis indicated that DHX34 expression correlated with clinicopathological features in a variety of tumors. The Protein-Protein Interaction (PPI) network and GSCALite database suggested that DHX34 and its ten co-expression genes might promote cancer progression by regulating the cell cycle. Gene Set Enrichment Analysis (GSEA) results further showed that DHX34 was positively correlated with pathways such as cell cycle, mitosis, and gene transcription regulation. The TISIDB database showed that DHX34 expression was closely associated with immune infiltration. Based on the TISCH2 database, we found that DHX34 was expressed in a number of immune cells, with relatively high expression in monocyte macrophages in LIHC.

**Conclusions:** In summary, our study found that DHX34 is highly expressed in pan-cancer and has diagnostic and prognostic value. Targeting DHX34 may improve the therapeutic efficacy of immunotherapy and chemotherapy in a multitude of tumors.

## Introduction

Cancer, a significant cause of mortality in the 21st century, is experiencing a rapid increase in both its incidence and mortality rates globally 1. Despite the clinical effectiveness of unconventional treatments such as radiotherapy, surgery, and chemotherapy, as well as advanced technologies including gene therapy, stem cell therapy, natural antioxidants, targeted therapy, photodynamic therapy, nanoparticles, and precision medicine, the prognosis for these patients remains unfavorable due to treatment resistance, side effects, and various other challenges 2-5. Therefore, it is crucial to develop novel biomarkers or therapeutic targets for cancer diagnosis and treatment.

The RNA helicase family, which is conserved from bacteria to humans, plays a pivotal role in every facet of RNA metabolism, including ribosome biogenesis, transcription, RNA maturation, the processing of MicroRNAs (miRNAs) and Circular RNAs (circRNAs), mRNA export, translation, and RNA degradation 6. Recent studies have unequivocally established the crucial role of the RNA helicase family in carcinogenic processes and immune modulation. Notably, DHX9 has been implicated in the tumorigenesis of various cancers 7. Remarkably, the deletion of DHX9 leads to a substantial reduction in cancer cell viability *in vitro* and fosters a significant boost in immunogenicity in mouse models of small-cell lung cancer, thereby greatly enhancing the responsiveness to immunotherapy 8. DHX15, another member of this family, is involved in the tumorigenesis of LIHC, gastric cancer, and colorectal cancer 9-11. Furthermore, DHX15 exhibits potential immune-regulatory effects by affecting the functions of dendritic cells, B cells, and NK cells 12-14. Additionally, DHX33 plays a pivotal role in the growth and proliferation of B-cells 15, and its overexpression in LIHC suggests its potential as a predictive biomarker for this cancer 16. Lastly, DHX37 exhibits a complex interaction with carcinogenesis, further underscoring the diverse and intricate roles of the RNA helicase family in cancer biology 17-19.

DHX34, a member of the DExD/H-box RNA helicase family, exhibits a profound connection with the onset of numerous diseases. For instance, its frequently altered splicing pattern has been observed in acute myeloid leukemia cases 20. Furthermore, the occurrence of preeclampsia is strongly linked to the methylation level of the DHX34 gene 21. Additionally, studies have reported that monoallelic variants of DHX34 are associated with neurodevelopmental disorders 22. Notably, DHX34 serves as a reliable predictor of LIHC prognosis within a prognostic risk score model 23. Despite these insights, however, there is no comprehensive study on the relationship between DHX34 and pan-cancer.

Therefore, the objective of our research was to investigate the expression levels of DHX34 and their association with diagnosis and prognosis across various cancer types. To achieve this, we utilized databases and platforms such as The Cancer Genome Atlas (TCGA), Gene Expression Omnibus (GEO), and Human Protein Atlas (HPA) database. Additionally, we conducted a comprehensive analysis to examine the mutational status, Protein-Protein Interaction (PPI) network, co-expression network, and biological functions of DHX34. Furthermore, we detected the relationships between DHX34 expression and various tumor characteristics, including TMB, MSI, Tumor Immune Microenvironment (TIME), Immune Checkpoint Inhibitors (ICI) response, and drug resistance. Our findings revealed that DHX34 exerts a pro-cancerous effect on cancer cells, indicating its potential as a diagnostic and prognostic biomarker in pan-cancer.

## Materials and methods

### Data acquisition and processing

From the TCGA database (https://portal.gdc.cancer.gov/), we retrieved RNA sequencing data and clinical follow-up information for patients with 33 distinct cancer types. This data allowed us to further explore the differential expression of DHX34 across various cancer subtypes. Additionally, we sourced GSE42568, GSE26566, GSE37182, GSE39791, GSE19804, and GSE71016 from the GEO database (https://www.ncbi.nlm.nih.gov/geo/) to complement our analysis of the expression of DHX34 in different cancer types. The "ggplot2" package in R software was employed for conducting comprehensive expression analysis and visualization.

The HPA database (https://www.proteinatlas.org/) provided us with information on the expression of DHX34 RNA and protein in human beings, and the DHX34 RNA expression in single-cell tissues and cancer cell lines. Also, the HPA database provides the subcellular localization of DHX34 using indirect immunofluorescence microscopy as well as visual representations of protein expression in human tissues after Immunohistochemistry (IHC) labeling 24.

### Patients and tissue samples

A total of 50 paraffin-embedded samples from LIHC cases underwent IHC staining. Furthermore, five pairs of frozen colonic carcinoma, LIHC, Lung Adenocarcinoma (LUAD), and Stomach Adenocarcinoma (STAD) tissues and their corresponding non-tumor tissues were utilized for Western blotting (WB) analysis. Comprehensive clinical data was collected for all patients. Following surgical intervention, all patients underwent regular follow-up procedures, including imaging scans and laboratory tests conducted every 3 to 6 months.

All tissues were obtained from the sample bank of the National and Local Joint Engineering Research Center of Biodiagnosis and Biotherapy at the Second Affiliated Hospital of Xi'an Jiaotong University. Before the commencement of this study, all patients had signed informed consent forms, and the research was approved by the ethics committee of the Second Affiliated Hospital of Xi'an Jiaotong University.

### Genomic alterations of DHX34 in pan‑cancer

The characteristics of genomic alterations in DHX34 across the pan-cancer were analyzed utilizing the cBioPortal database (https://www.cbioportal.org) 25. This comprehensive analysis focused on investigating the genetic alteration rate, mutation types, and specific mutated site information of DHX34 in pan-cancer.

### Correlation of DHX34 expression with MSI and TMB in pan‑cancer

The TMB and MSI scores were sourced from the TCGA database. Subsequently, Spearman's correlation analysis was conducted to evaluate the associations between the expression levels of DHX34 and both TMB and MSI.

### The diagnostic and prognostic value of DHX34 in pan‑cancer

To assess the diagnostic potential of DHX34 in 33 different cancer types, Receiver Operating Characteristic (ROC) curves were employed. The analysis and visualization of these data were facilitated by the "pROC" and "ggplot2" packages in R software. To analyze the relationship between DHX34 expression and the prognosis of these cancers, we focused on three key metrics: Overall Survival (OS), Disease-Specific Survival (DSS), and Progression-Free Interval (PFI). We assessed the correlation of DHX34 expression with OS, DSS, and PFI using univariate Cox regression analysis using the survival package and visualized using the "ggplot2" package. Subsequently, Patients were classified into high and low DHX34 expression groups based on the median DHX34 expression in different cancers. We performed survival analyses using the "survival" packages in R software to detect the link between DHX34 and survival prognosis.

### The correlation between DHX34 expression and clinicopathological features in pan‑cancer

To elucidate potential correlations between DHX34 expression and various clinicopathologic indicators across pan-cancer, we employed the Wilcoxon or Kruskal-Wallis test. These indicators encompassed pathologic T stage, pathologic stage, pathologic M stage, WHO grade, IDH status, AFP levels, histologic grade, and radiation therapy. Furthermore, to gain a deeper understanding of the relationship between DHX34 and specific clinical parameters in LIHC, we utilized the chi-square test and logistic regression analysis.

### The related genes and PPI Network analysis of DHX34 in pan‑cancer

We respectively analyzed the 20 genes with the highest correlation to DHX34 across the 8 tumors in which DHX34 has a prognostic value and visualized them using the "ggplot2" package. Additionally, We analyzed the PPI network of DHX34 using the STRING database (https://cn.string-db.org/) 26. The top 10 genes with the highest correlation in the co-expression network in LIHC and the top 10 genes with the highest interaction score in the PPI network were selected. we utilized the Tumor Immune Estimation Resource 2.0 (TIMER2) (http://timer.cistrome.org/) 27 to examine the correlations between DHX34 and its related genes across pan-cancer. We performed correlation analysis of the above genes in LHC and visualized them using the "circlize" package.

### Prognostic value and functional analysis of DHX34-related genes

We analyzed the prognostic value of the above 20 genes in LIHC using "survival" packages. In addition, we analyzed the signaling pathways regulated by DHX34 and its related genes with prognostic value in LIHC using GSCALite (https://guolab.wchscu.cn/GSCA) 28.

### The Differentially Expressed Gene and Gene Set Enrichment Analysis (GSEA) analysis of DHX34 in pan‑cancer

We divided the patients into high and low expression groups based on the median expression level of DHX34 and analyzed the differential genes using the "DESeq2" package, visualizing them in the "ggplot2" package. The genes displayed are:|log2(FC)| > 1.5 and a p < 0.05. To ascertain the biological pathway variations between high- and low-DHX34 groups, the "clusterProfiler" package performed the GSEA analysis. The False Discovery Rate (FDR) < 0.25 and an adjusted p-value < 0.05 were regarded as remarkable altered pathways.

### The correlation between DHX34 expression and the TIME in pan-cancer

The relationship between DHX34 expression and immune system-related modulators in various malignancies was evaluated using the TISIDB online database (http://cis.hku.hk/TISIDB/index.php) 29. These modulators included Tumor-Infiltrating Lymphocytes (TIL), immune stimulators, immune inhibitors, Major Histocompatibility Complex (MHC), chemokine, and receptors.

### The single-cell expression analysis of DHX34 in LIHC

To determine the possible function of DHX34 at the single-cell level, the relationship between DHX34 expression and immune cells was examined using the Tumor Immune Single-cell Hub 2 (TISCH2) database (http://tisch.comp-genomics.org/) 30.

### The immunotherapy and chemotherapy response analysis of DHX34 in pan-cancer

From the TCGA dataset, RNA-sequencing expression (level 3) profiles and related clinical data for pan-cancer were retrieved. Subsequently, the Tumor Immune Dysfunction and Exclusion (TIDE) method was employed to predict the potential response to ICI treatment 31. This analysis was facilitated by "ggplot2" and "ggpubr" packages in R software. We examined the relationship between DHX34 expression and drug sensitivity for pan-cancer using the GSCALite 28.

### Correlation analysis of DHX34 with ferroptosis and m6A-related genes in LIHC

Ferroptosis refers to the impaired metabolism of intracellular lipid oxides and the production of toxic lipids to induce cell death, m6A is RNA methylation, a methylation on the 6th N atoms on adenine (A) in RNA that affects mRNA stability, translation efficiency, variable splicing, and localization. We analyzed the correlation of DHX34 with Ferroptosis and m6A-related genes in LIHC. Ferroptosis-related genes were derived from Ze-Xian Liu *et al.* Systematic analysis of the abnormalities and functions of iron death in cancer 32. The m6A-associated genes were derived from a study by Juan Xu *et al.* on the molecular characterization and clinical significance of m6A regulators across 33 cancer types 33.

### RNA preparation and Quantitative Real-Time PCR (qRT-PCR)

Total RNA was extracted from tissues using the TRIZOL reagent (Invitrogen) by the manufacturer's instructions. Using a PrimeScript RT Reagent Kit (Takara), the purified RNA was converted to cDNA. qRT-PCR tests were conducted using the Takara SYBR Premix Ex Taq II Kit. The results were adjusted to the expression of GAPDH. The primer sequences utilized in this investigation were as follows:

GAPDH-forward: TGTGGGCATCAATGGATTTGG

GAPDH-reverse: ACACCATGTATTCCGGGTCAAT

DHX34-forward: TGAGAGCCTCAGTCAGTATGG

DHX34-reverse: TGTCAGGAATACAATCTTGGTGG

### Western Blotting

Tissues were lysed in RIPA buffer (Beyotime Biotechnology, China) containing a protease inhibitor cocktail. Following the use of a BCA assay kit (Beyotime, Jiangsu, China) to measure the concentration of protein, equal amounts of protein were separated using 10% Sodium Dodecyl Sulfate Polyacrylamide Gel Electrophoresis (SDS-PAGE) and then deposited onto a Polyvinylidene Difluoride (PVDF) membrane. Specific primary antibodies were used to incubate the proteins, including anti-DHX34 (1:1000, Affinity) and anti-GAPDH (1:2000, CST). Following three rounds of washing, the membrane was left to be incubated for two hours at room temperature with secondary antibodies that matched its species. Lastly, protein visualization was performed using the Enhanced Chemiluminescence (ECL) Western Blot Detection Kit (Millipore). The protein loading control was GAPDH.

### Immunohistochemistry

The tumor and normal tissues fixed in paraffin were sectioned at a thickness of 4 µm.

After deparaffinizing and hydrating, these sections were then subjected to a heat treatment at 95 °C within a citric acid buffer adjusted to a pH of 6.0, aiming to extract the antigens. Before incubation with the primary antibodies, the slices were treated with 3% H2O2 and subsequently blocked with 5% goat normal serum. The primary antibodies against DHX34 (1:200, Affinity) were applied, followed by the appropriate secondary antibody. Next, the sections were visualized with Diaminobenzidine (DAB) and finally counterstained with Hematoxylin. We performed a semi-quantitative analysis using Image-Pro Plus 6.0 software by capturing five random microscopic images of each section. The analysis encompassed the area and density of the stained region, and Integrated Optical Density (IOD). The average of five IOD values per section served as a reliable indicator to reflect DHX34 expression levels.

### Statistical analysis

The aforementioned packages in R version 4.0.3 and Graphpad Prism 8.0 were used to analyze and visualize the data. The Welch one-way ANOVA was used to evaluate comparisons between several groups. The Student t-test was employed to evaluate comparisons between the two groups. Each experiment was performed thrice and data were shown as mean ± Standard Deviation (SD). Any value of p<0.05 was considered to be statistically significant.

## Results

### The expression of DHX34 in human organs and tissues

**Figure 1:**
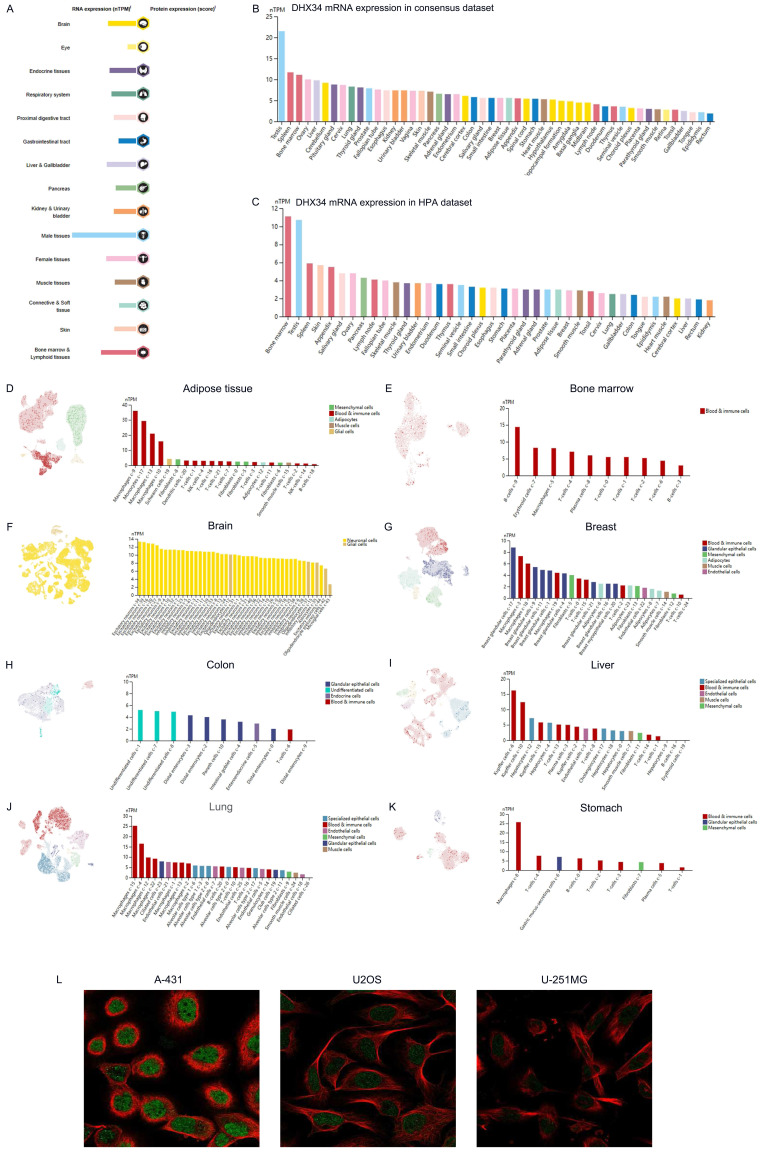
The expression of DHX34 in human organs and tissues. (A) Overview of DHX34 mRNA and protein expression across human organs and tissues. (B, C) Summarized DHX34 mRNA expression in various organs and tissues, based on the consensus and HPA dataset. (D-K) Expression analysis of DHX34 mRNA in distinct single cell tissues. (L) The subcellular localization of DHX34, as depicted by immunofluorescence visualization in HPA database.

The mRNA of DHX34 was widely expressed in various human organs and tissues (Fig. 1A). Analysis of the consensus dataset revealed that DHX34 mRNA is primarily expressed in the testis, spleen, bone marrow, ovary, liver, cerebellum, pituitary gland, cervix, lung, and thyroid gland (Fig. 1B). Furthermore, data acquired from the HPA database indicated that DHX34 is predominantly expressed in bone marrow, testis, spleen, skin, appendix, salivary gland, ovary, pancreas, lymph node, and fallopian tube (Fig. 1C). The detailed expression patterns of DHX34 in various single-cell tissues, including adipose tissue, bone marrow, brain, breast, colon, liver, lung and stomach were shown in Fig. 1D-K. Moreover, we obtained DHX34 subcellular localization from the HPA database. DHX34 subcellular localization was obtained by immunofluorescence localization of the nuclei, microtubules, and ER in A-431, U-2OS, and U-251MG cells, the green color represents the location and intensity of DHX34 expression, which shows that DHX34 was primarily located in the nucleoplasm (Fig. 1L). These three cells are indispensable in tumor research and are widely used in cell biology and molecular biology studies. Based on the importance and representativeness of these three cells, we chose them to study the subcellular localization of DHX34. In addition, these three cells are relatively easy to culture in experimental manipulation, which can ensure the accuracy and reproducibility of experimental results.

### DHX34 is highly expressed in most tumors

**Figure 2:**
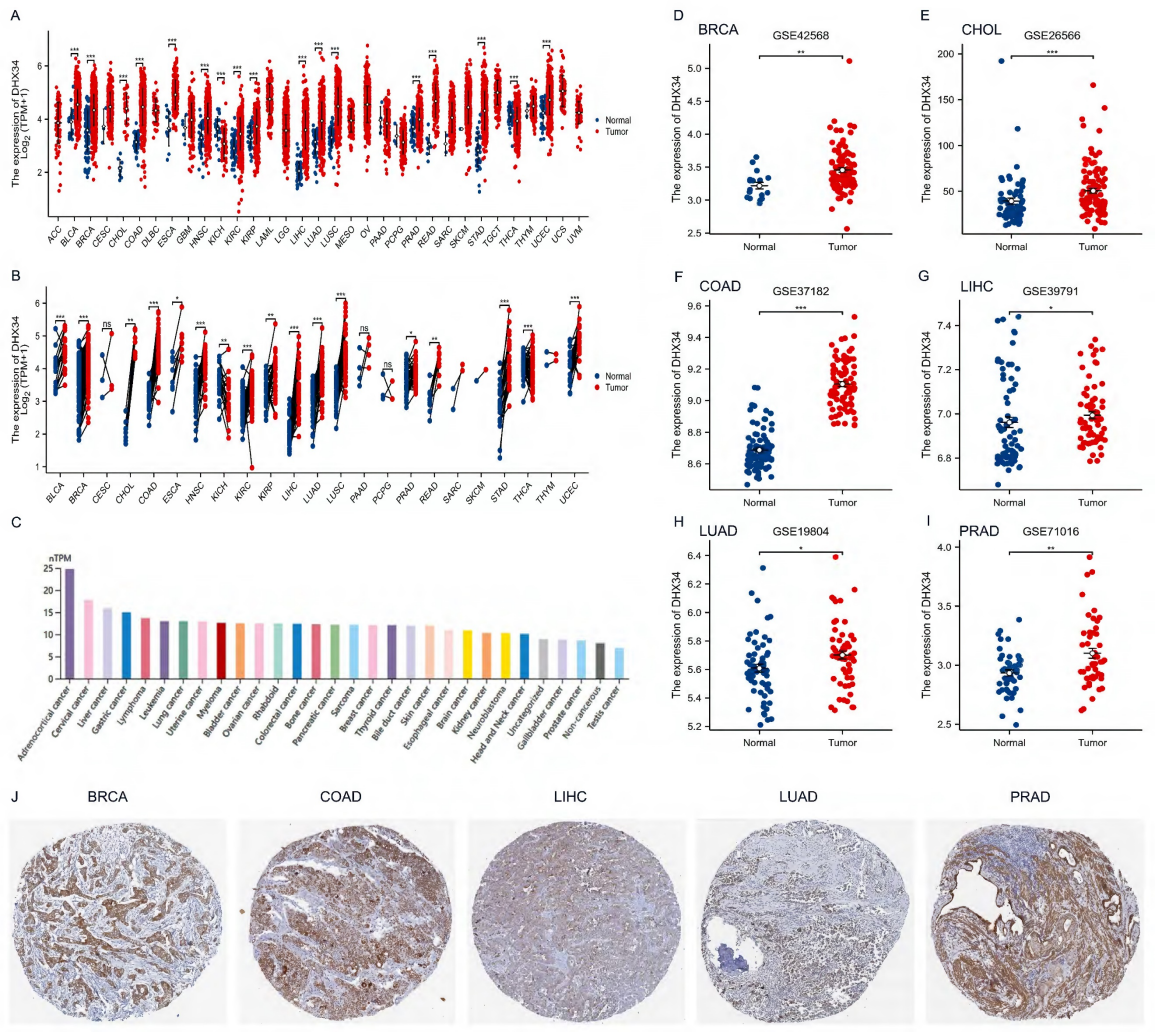
The expression of DHX34 in Pan-Cancer. (A, B) The mRNA expression of DHX34 in unpaired and paired pan-cancerous tissues, as depicted in the TCGA database. (C) The mRNA expression of DHX34 in different cancer cell lines. (D-I) The mRNA expression of DHX34 in BRCA, CHOL, COAD, LIHC, LUAD, and PRAD, based on data from the GEO database. (J) The protein expression of DHX34 in BRCA, COAD, LIHC, LUAD and PRAD by IHC staining from the HPA database. (ns: no significance; * p < 0.05; ** p < 0.01; *** p < 0.001).

Upon analyzing pan-cancer data from TCGA, we discovered an upregulation of DHX34 expression in both unpaired and paired tumor tissues across 15 cancer types, including Bladder Urothelial Carcinoma (BLCA), Breast Invasive Carcinoma (BRCA), Cholangiocarcinoma (CHOL), Colon Adenocarcinoma (COAD), Esophageal Carcinoma (ESCA), Head and Neck Squamous Cell Carcinoma (HNSC), Kidney Renal Clear Cell Carcinoma (KIRC), Kidney Rrenal Papillary Cell Carcinoma (KIRP), Liver Hepatocellular Carcinoma(LIHC), Lung Adenocarcinoma (LUAD), Lung Squamous Cell Carcinoma (LUSC), Prostate Adenocarcinoma (PRAD), Rectum Adenocarcinoma (READ), Stomach Adenocarcinoma (STAD) and Uterine Corpus Endometrial Carcinoma (UCEC) (Fig. 2A, 2B). Furthermore, analysis of the HPA dataset revealed that DHX34 mRNA is mainly expressed in adrenocortical cancer, cervical cancer, and liver cancer cell lines (Fig. 2C). Consistent with these findings, our evaluation of six GEO datasets indicated overexpression of DHX34 in BRCA, CHOL, COAD, LIHC, LUAD, and PRAD (Fig. 2D-I). To further validate the protein expression pattern of DHX34, we examined IHC-staining images from the HPA database, which highlighted the elevated expression of DHX34 in BRCA, COAD, LIHC, LUAD, and PRAD (Fig. 2J).

### The gene mutation of DHX34 in pan-cancer

**Figure 3:**
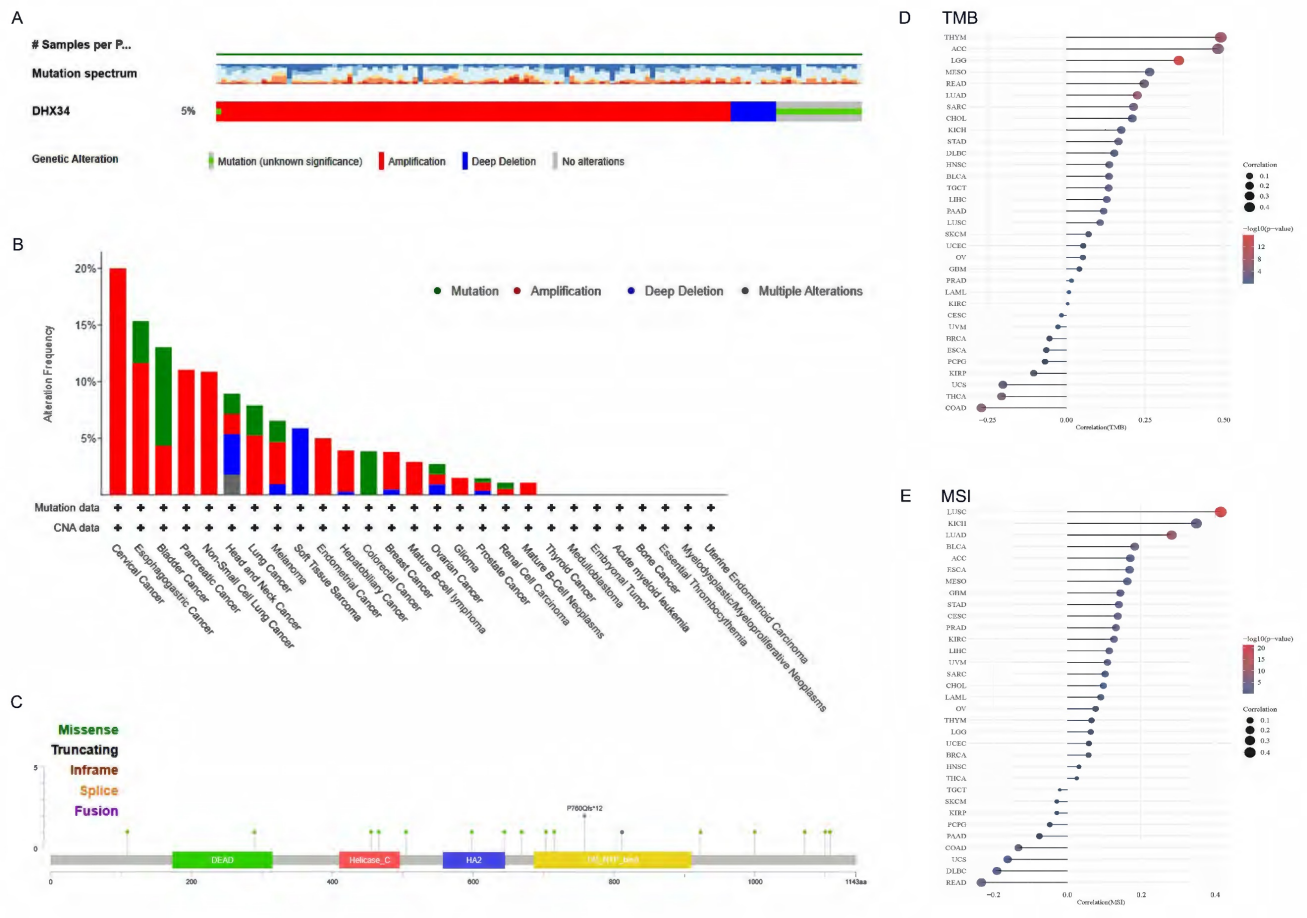
The genetic alterations of DHX34 in Pan-Cancer. (A) The genetic alteration profile of DHX34 in pan-cancer. (B) The genetic alteration frequencies of DHX34 in pan-cancer. (C) The mutation sites of DHX34 in pan-cancer. (D, E) The correlation between DHX34 expression and TMB as well as MSI according to TCGA database.

To assess the mutation of DHX34 in pan-cancer, we conducted a comprehensive study using the cBioPortal database and found that DHX34 was altered in 5% (128/2565) of pan-cancer patients (Fig. 3A). Furthermore, our analysis of the mutation frequency of the DHX34 gene across various tumor types showed that cervical cancer (20%), esophagogastric cancer (15.34%), and bladder cancer (13.04%) had the highest alteration frequency, ranking among the top three. Notably, amplification was identified as the most prevalent type of DHX34 gene mutation (Fig. 3B). Analysis of the mutation sites of DHX34 in pan-cancer, revealed a total of 18 mutation sites, spanning the region between 0 and 1143 amino acids (Fig. 3C). Additionally, a positive correlation was observed between DHX34 expression and TMB across 12 tumor types, and MSI across10 tumor types (Fig. 3D, 3E), indicating that DHX34 significantly impacts both TMB and MSI.

### The diagnostic value of DHX34 in pan-cancer

**Figure 4:**
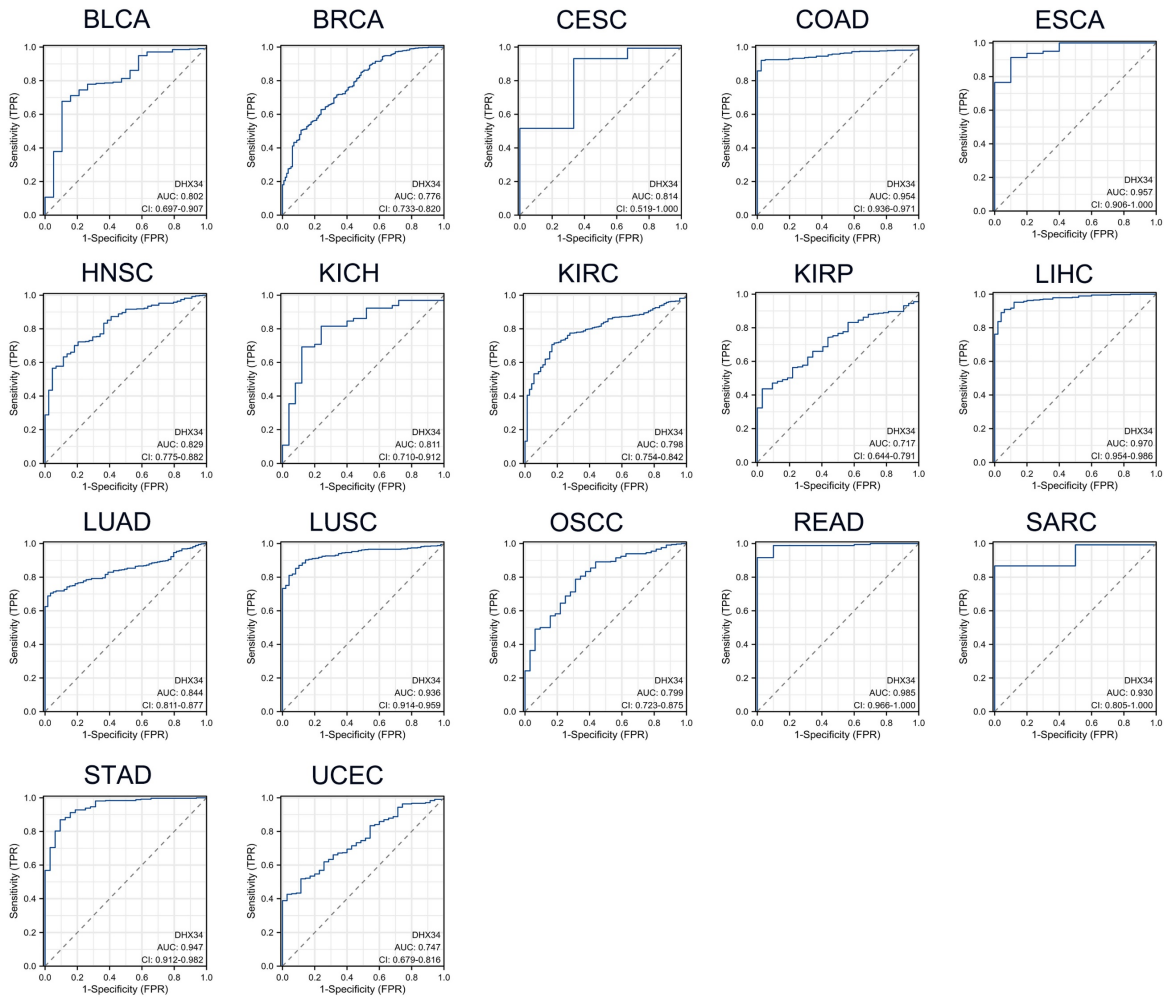
The diagnostic values of DHX34 in Pan-Cancer. (A-R) ROC curves were used to predict the diagnostic value of DHX34 in pan-cancer.

As shown in Fig. 4, DHX34 has a good diagnostic value in a variety of cancers, including BLCA (AUC = 0.802, 95% CI: 0.697-0.907), BRCA (AUC = 0.776, 95% CI: 0.733-0.820), Cervical Squamous Cell Carcinoma and Endocervical Adenocarcinoma (CESC) (AUC = 0.814, 95% CI: 0.519-1.000), COAD (AUC = 0.954, 95% CI: 0.936-0.971), Esophageal Carcinoma (ESCA) (AUC = 0.957, 95% CI: 0.906-1.000), HNSC (AUC = 0.829, 95% CI: 0.775-0.882), Kidney Chromophobe (KICH) (AUC = 0.811, 95% CI: 0.710-0.912), KIRC (AUC = 0.798, 95% CI: 0.754-0.842), KIRP (AUC = 0.717, 95% CI: 0.644-0.791), LIHC (AUC = 0.970, 95% CI: 0.954-0.986), LUAD (AUC = 0.844, 95% CI: 0.811-0.877), LUSC (AUC = 0.936, 95% CI: 0.914-0.959), Oral Squamous Cell Carcinoma (OSCC) (AUC = 0.799, 95% CI: 0.723-0.875), READ (AUC = 0.985, 95% CI: 0.966-1.000), Sarcoma (SARC) (AUC = 0.930, 95% CI: 0.805-1.000), STAD (AUC = 0.947, 95% CI: 0.912-0.982), UCEC (AUC = 0.747, 95% CI: 0.679-0.816).

### The prognostic value of DHX34 in pan-cancer

**Figure 5:**
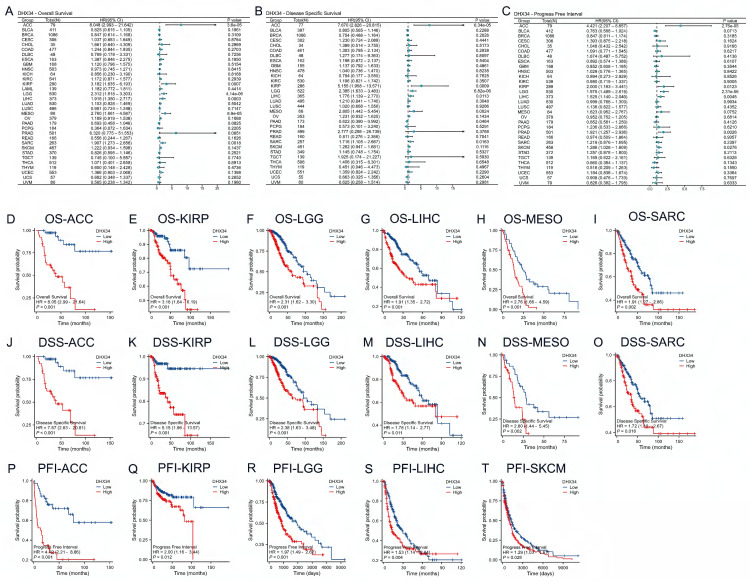
The prognostic values of DHX34 in Pan-Cancer. (A) The forest plot shows the univariate Cox regression analysis results of DHX34 on OS in TCGA pan-cancer, (B) The forest plot shows the univariate Cox regression analysis results of DHX34 on DSS in TCGA pan-cancer, (C) The forest plot shows the univariate Cox regression analysis results of DHX34 on PFI in TCGA pan-cancer, Correlations of DHX34 with OS (D-I), DSS (J-O), and PFI (P-T) in pan-cancer.

To investigate the prognostic value of DHX34, we performed univariate Cox regression analysis to evaluate DHX34 expression with OS, DSS, and PFI in pan-cancer. Forest map showing the prognostic value of DHX34 in a variety of cancer types (Fig. 5A-C). To further determine the prognostic value of DHX34, survival analysis was performed. Our findings revealed that high expression of DHX34 was significantly correlated with shorter OS in Adrenocortical Carcinoma (ACC) (HR = 8.05, 95% CI: 2.99-21.64, p < 0.001), KIRP (HR = 3.18, 95% CI: 1.64-6.19, p < 0.001), Low-Grade Glioma (LGG) (HR = 2.31, 95% CI: 1.62-3.30, p < 0.001), LIHC (HR = 1.91, 95% CI: 1.35-2.72, p < 0.001), Malignant Mesothelioma (MESO) (HR = 2.76, 95% CI:1.66-4.59, p < 0.001), SARC (HR = 1.91, 95% CI: 1.27-2.86, p = 0.002) (Fig. 5D-I). Additionally, a significant association was observed between high DHX34 expression and shorter DSS in ACC (HR = 7.67, 95% CI: 2.83-20.81, p < 0.001), KIRP (HR = 5.15, 95% CI: 1.96-13.57, p < 0.001), LGG (HR = 2.38, 95% CI: 1.63-3.48, p < 0.001), LIHC (HR = 1.78, 95% CI: 1.14-2.77, p = 0.011), MESO (HR = 2.80, 95% CI: 1.44-5.45, p = 0.002), SARC (HR = 1.72, 95% CI: 1.10-2.67, p = 0.016) (Fig. 5J-O). Furthermore, high DHX34 expression was associated with shorter PFI in ACC (HR = 4.42, 95% CI: 2.21-8.86, p < 0.001), KIRP (HR = 2.00, 95% CI: 1.16-3.44, p = 0.012), LGG (HR = 1.97, 95% CI: 1.49-2.62, p < 0.001), LIHC (HR = 1.53, 95% CI: 1.14-2.04, p = 0.004), and Skin Cutaneous Melanoma (SKCM) (HR = 1.29, 95% CI: 1.03-1.61, p = 0.028) (Fig. 5P-T).

### The correlation between DHX34 expression and clinicopathological characteristics

**Figure 6:**
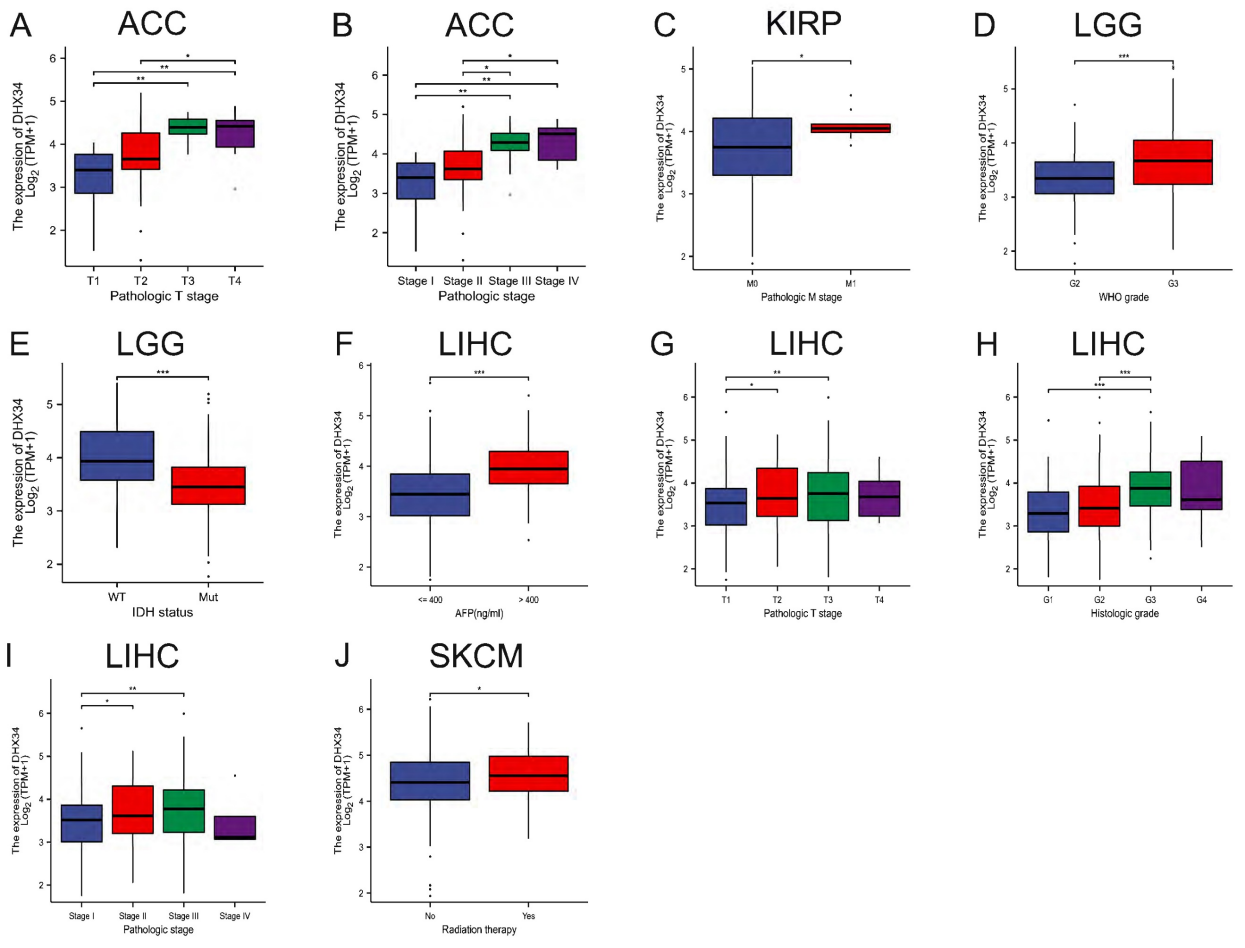
The correlations of DHX34 expression and clinical features in pan-cancer. (A, B) The correlations of DHX34 expression with pathologic T stage and pathologic stage in ACC. (C) The correlations of DHX34 expression with pathologic M stage in KIRP. (D, E) The correlations of DHX34 expression with WHO grade and IDH status in LGG. (F-I) The correlations of DHX34 expression with AFP, pathologic T stage, histologic grade, and pathologic stage in LIHC. (J) The correlations of DHX34 expression with radiation therapy in SKCM. (* *p* < 0.05; ** p < 0.01; *** *p* < 0.001).

In a subgroup analysis, we observed that high DHX34 expression correlated with advanced pathologic T stage and pathologic stage in ACC (Fig. 6A, 6B). Similarly, it was correlated with the pathologic M stage in KIRP (Fig. 6C). Furthermore, high DHX34 expression was associated with higher WHO grade and IDH status (WT) in LGG (Fig. 6D, 6E). In LIHC, high AFP levels, pathologic T stage, histologic grade, and pathologic stage were all found to be correlated with high DHX34 expression (Fig. 6F-I). Lastly, patients who underwent radiation therapy displayed a correlation with high DHX34 expression in SKCM (Fig. 6J).

We employed the logistic regression method to analyze the link between DHX34 expression levels and the clinicopathologic characteristics of LIHC. The findings indicated a strong association between DHX34 expression and gender (P = 0.036), Age (p = 0.044), AFP (ng/mL) (p < 0.001), prothrombin time (p = 0.031), and histologic grade (p < 0.001) (Table 1).

### Identification of DHX34-related genes and PPI network

**Figure 7:**
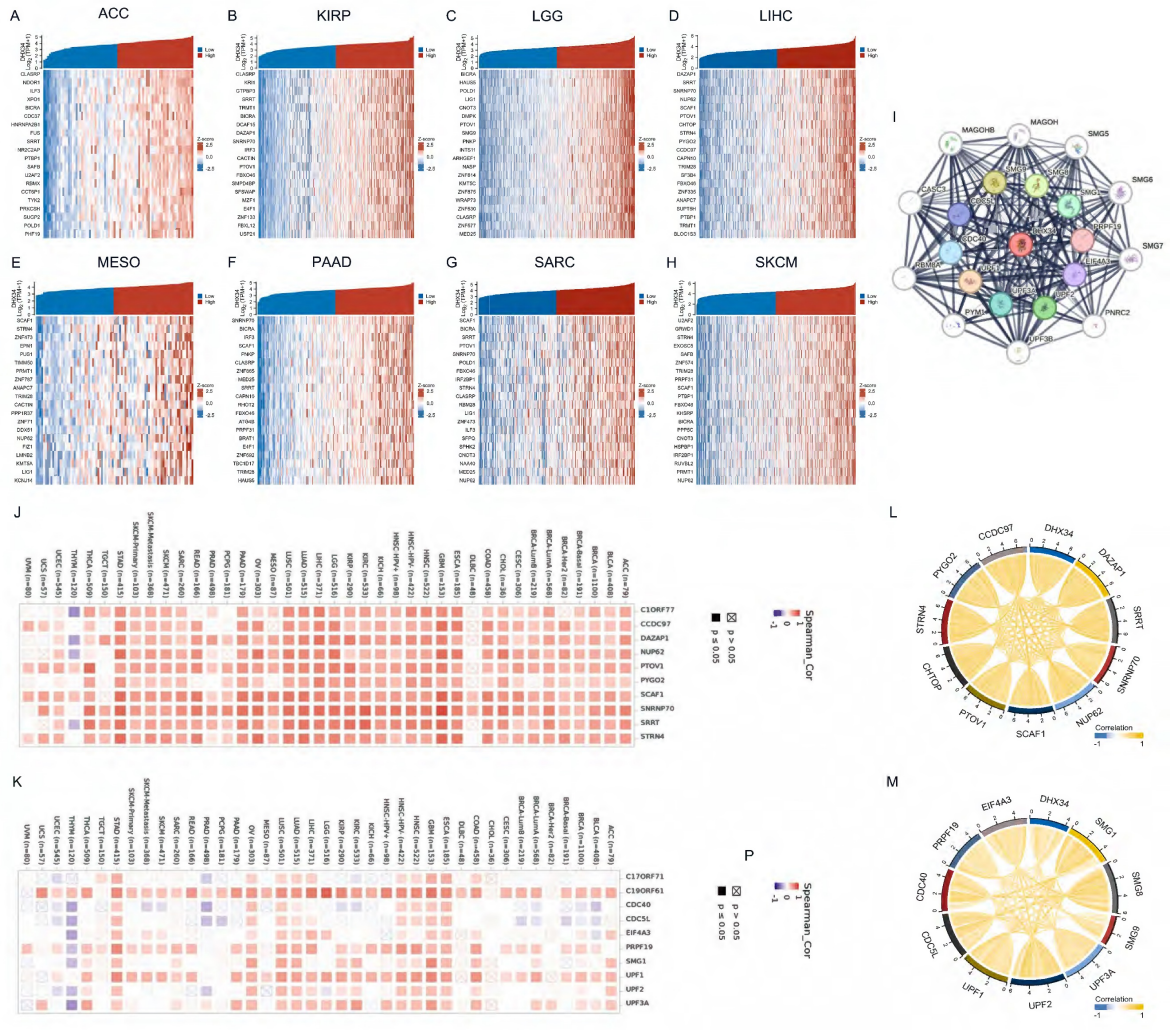
DHX34-related genes and PPI Network. (A-H) Heatmap of top 20 co-expressed genes of DHX34 in ACC, KIRP, LGG, LIHC, MESO, PAAD, SARC, SKCM, (I) The top 20 DHX34-related proteins via PPI network analysis, (J-K) Heatmap of the top 10 correlations of co-expression network and the PPI network in the pan-cancer, (L-M) Correlation of each of the top 10 genes in LIHC.

We analyzed the co-expressed genes of DHX34 in eight cancer types using RNA sequencing data obtained from the TCGA database and visualized the 20 most highly correlated genes (Fig. 7A-H). Using the STRING tool, we identified the top 20 proteins that interact with DHX34 (Fig. 7I). Following this, we utilized TIMER2 to investigate the co-expression patterns of the 10 most highly correlated genes in LIHC and the top 10 genes with the highest interaction score. The results indicated that most of these genes displayed a positive correlation with DHX34 in pan-cancer (Fig. 7J-K). Finally, we analyzed the correlations between the above genes, and the results showed that all of these genes were significantly correlated with each other (Fig. 7L-M).

### DHX34-related genes of prognostic value and functional pathway

**Figure 8:**
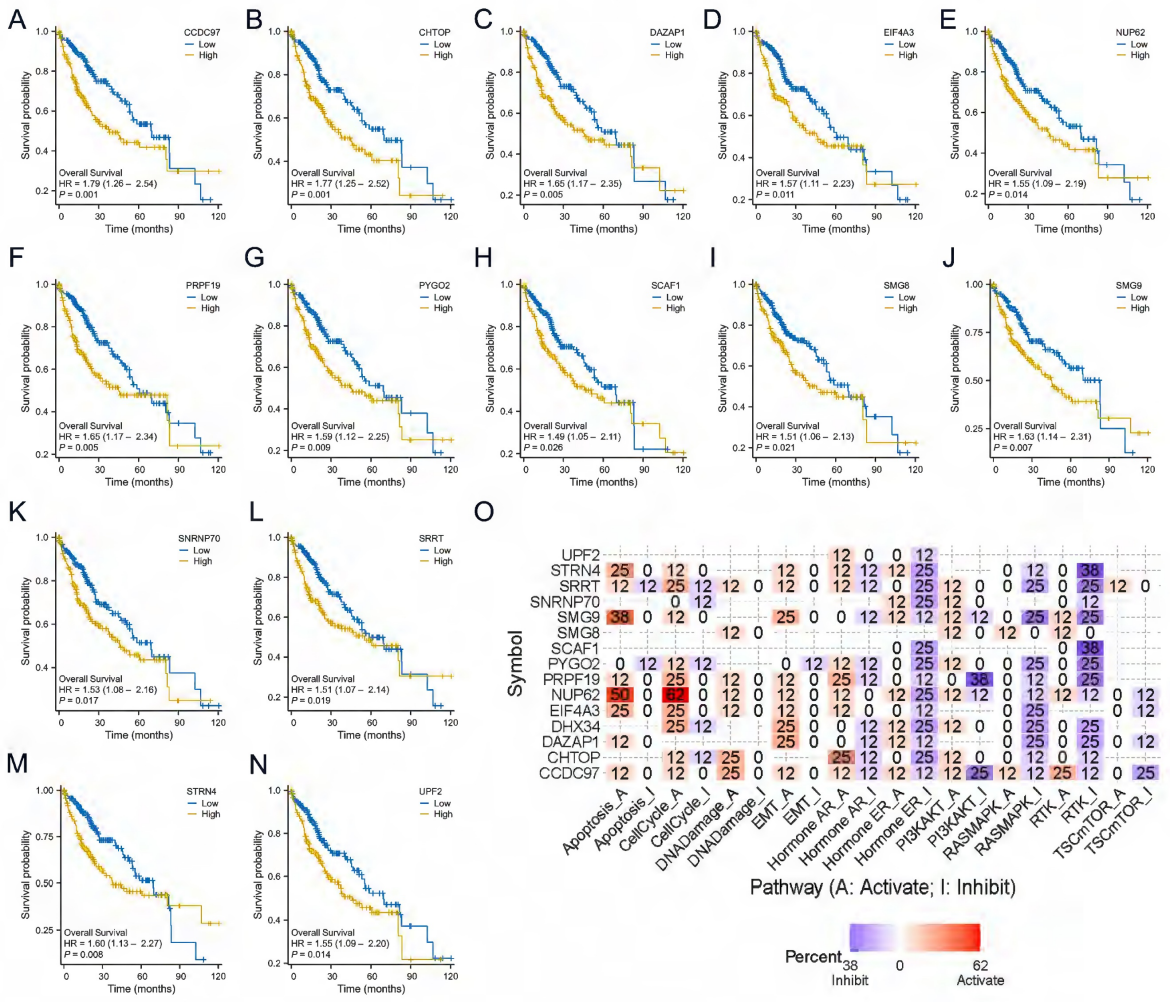
DHX34-related genes of prognostic value and Functional pathway. (A-N) Prognostic value of CCDC97, CHTOP, DAZAP1, EIF4A3, NUP62, PRPF19, PYGO2, SCAF1, SMG8, SMG9, SNRNP70, SRRT, STRN4, UPF2 in LIHC. (O) DHX34-related genes Potential Common functional pathway.

We analyzed the prognostic value of the above 20 genes and showed that 14 genes had prognostic value in LIHC, CCDC97 (HR=1.79, CI:1.26-2.54, p=0.001), CHTOP (HR=1.77, CI:1.25-2.52, p=0.001), DAZAP1 (HR=1.65, CI:1.17-2.35, p=0.005), EIF4A3 (HR=1.57, CI:1.11-2.23, p=0.011), NUP62 (HR=1.55, CI:1.09-2.19, p=0.014), PRPF19 (HR=1.65, CI:1.17-2.34, p=0.005), PYGO2 (HR=1.59, CI:1.12-2.25, p=0.009), SCAF1 (HR=1.49, CI:1.05-2.11, p=0.026), SMG8 (HR=1.51, CI:1.06-2.13, p=0.021), SMG9 (HR=1.63, CI:1.14-2.31, p=0.007), SNRNP70 (HR=1.53, CI:1.08-2.16, p=0.017), SRRT (HR=1.51, CI:1.07-2.14, p=0.019), STRN4 (HR=1.60, CI:1.13-2.27, p=0.008), UPF2 (HR=1.55, CI:1.09-2.20, p=0.014)(Fig. 8A-N). We leveraged the GSCALite to examine the potential roles of DHX34 and these 14 genes in LIHC, which suggests that these genes may promote the progression of LIHC by modulating the apoptosis and cell cycle (Fig. 8O).

### The DEGs and GSEA enrichment analysis of DHX34 in pan‑cancer

**Figure 9:**
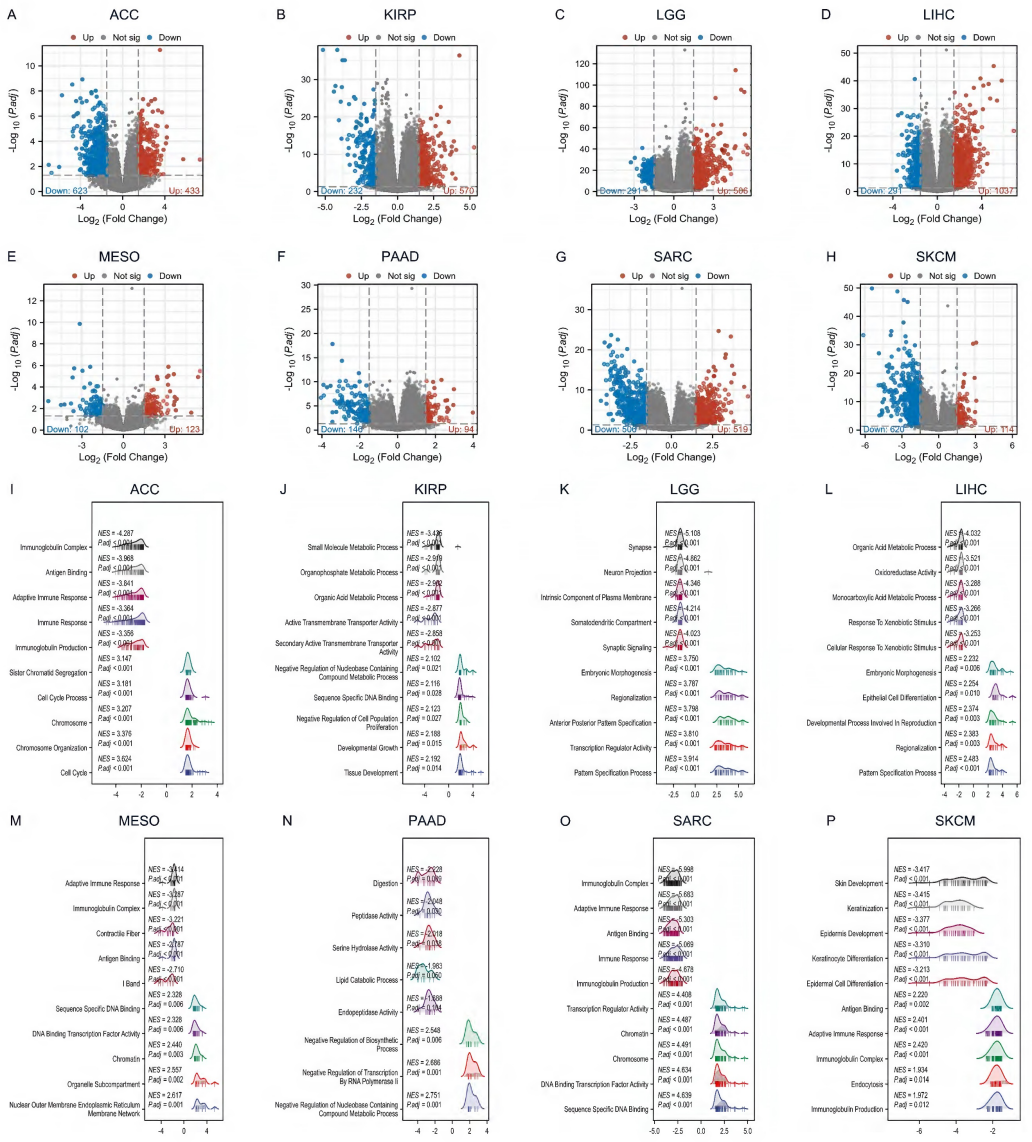
The DEGs and GSEA enrichment analysis of DHX34 in Pan‑Cancer. (A-H) The differential gene volcano map of DHX34 in ACC, KIRP, LGG, LIHC, MESO, PAAD, SARC, SKCM, (I-P) The GSEA functional enrichment pathways of DHX34 in ACC, KIRP, LGG, LIHC, MESO, PAAD, SARC, SKCM.

By differential gene analysis, we found a large number of differential genes in DHX34 in all eight tumors, ACC (433 up-regulated genes and 623 down-regulated genes), KIRP (570 up-regulated genes and 232 down-regulated genes), LGG (506 up-regulated genes and 291 down-regulated genes), LIHC (1037 up-regulated genes and 291 down-regulated genes), MESO (123 up-regulated genes and 102 down-regulated genes), PAAD (94 up-regulated genes and 146 down-regulated genes), SARC (519 up-regulated genes and 506 down-regulated genes), SKCM (114 up-regulated genes and 620 down-regulated genes) (Fig. 9A-H).

To determine the DHX34-associated KEGG pathways, we conducted a GSEA. Our results revealed that in ACC, DHX34 was positively associated with the cell cycle (NES = 3.624, P.adj < 0.001), and negatively associated with immunoglobulin complex (NES = -4.287, P.adj < 0.001) (Fig. 9I). In KIRP, DHX34 showed positive associations with tissue development (NES = 2.192, P.adj = 0.014) and negative associations with small molecule metabolic process (NES = -3.435, P.adj < 0.001) (Fig. 9J). In LGG, DHX34 positively correlated with the pattern specification process (NES = 3.914, P.adj < 0.001) and negatively with a synapse (NES = -5.108, P.adj < 0.001) (Fig. 9K). For LIHC, DHX34 displayed positive associations with the pattern specification process (NES = 2.483, P.adj < 0.001) and negative associations with the organic acid metabolic process (NES = -4.032, P.adj < 0.001) (Fig. 9L). In MESO, DHX34 positively correlated with nuclear outer membrane endoplasmic reticulum membrane network (NES = 2.617, P.adj = 0.001) and negatively with adaptive immune response (NES = -3.414, P.adj < 0.001) (Fig. 9M). In PAAD, DHX34 showed positive associations with Negative Regulation of Nucleobase Containing Compound Metabolic Process (NES = 2.751, P.adj = 0.001) and negative associations with Digestion (NES = -2.228, P.adj = 0.009) (Fig. 9N). For SARC, DHX34 showed positive associations with sequence-specific DNA binding (NES = 4.639, P.adj < 0.001) and negative associations with immunoglobulin complex (NES = -5.998, P.adj < 0.001) (Fig. 9O). Finally, in SKCM, DHX34 positively correlated with immunoglobulin production (NES = 1.972, P.adj = 0.012) and negatively with skin development (NES = -3.417, P.adj < 0.001) (Fig. 9P). These findings indicate that DHX34 is extensively involved in regulating cellular biological functions across multiple cancer types.

### Correlation of DHX34 with TIME in pan‑cancer

**Figure 10:**
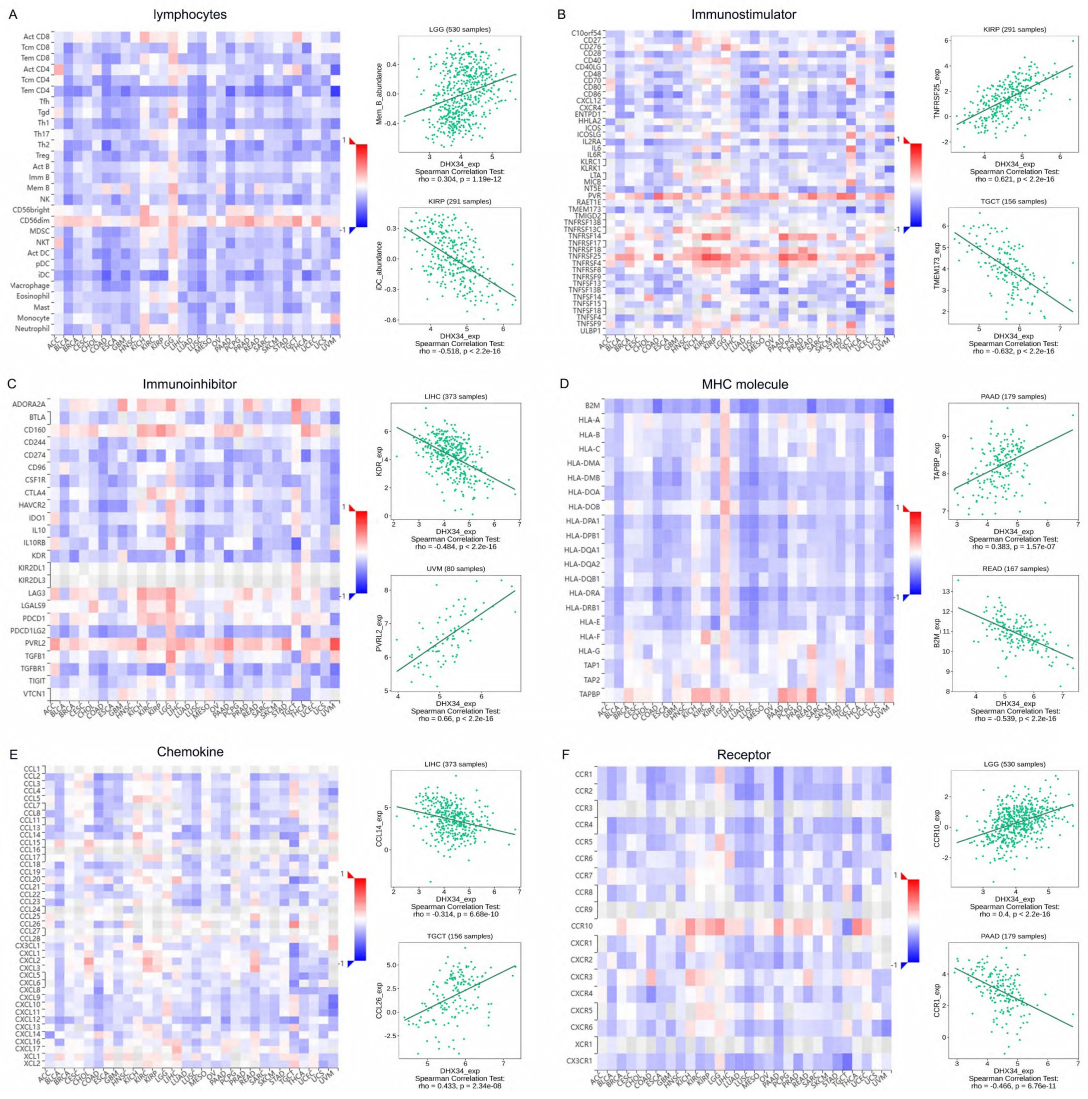
Correlations of DHX34 expression with the expression of immunomodulators. A-F Correlations between the expression of DHX34 and (A) lymphocyte, (B) immune stimulator, (C) immune inhibitor, (D) MHC molecules, (E) chemokine, and (F) receptor in the TISIDB database. The red and blue represent positive and negative correlations, respectively.

We conducted gene co-expression analyses in the TISIDB database to explore the relationship between the expression of DHX34 and various components of TIME, including lymphocytes, immune stimulators, immune inhibitors, MHC molecules, chemokines, and receptors. Our study revealed significant correlations between DHX34 expression and multiple immune factors in pan-cancer. Specifically, DHX34 expression showed a positive correlation with the expression of lymphocyte subsets such as Mem B in LGG and a negative correlation with iDC in KIRP (Fig. 10A). Among the 45 immune stimulators studied, DHX34 expression positively correlated with TNFRSF25 in KIRP and negatively correlated with TMEM173 in TGCT (Fig. 10B). In the analysis of 24 immune inhibitors, we observed a negative association between DHX34 expression and KDR in LIHC, while a positive association was found between DHX34 expression and PVRL2 in UVM (Fig. 10C). Fig. 10D demonstrated that DHX34 expression positively correlated with TAPBP in PAAD and negatively correlated with MHC molecule B2M in READ. Additionally, our study of chemokines revealed a negative correlation between DHX34 expression and CCL14 in LIHC, while a positive correlation was observed between DHX34 expression and CCL26 in TGCT (Fig. 10E). In the analysis of receptors, DHX34 expression positively correlated with CCR10 in LGG and negatively correlated with CCR1 in PAAD (Fig. 10F). Collectively, these findings indicated that DHX34 holds promising potential in predicting immune-related phenotypes in pan-cancer.

### The single-cell expression of DHX34 in LIHC

**Figure 11:**
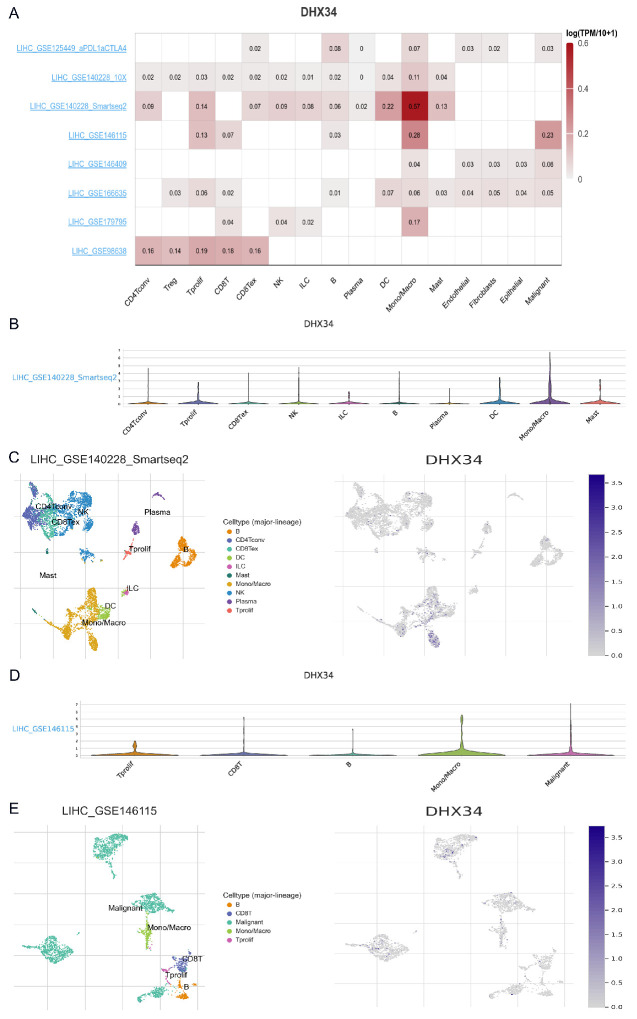
The single cell expression of DHX34 in LIHC. (A) Heatmap of the independent scRNA-seq database showing the expression level of DHX34 in different kinds of immune cells. (B) Violin plot of the expression level of DHX34 in immune cells in LIHC_GSE140228 Smartseq2 Dataset. (C) The cell type distribution and DHX34 expression in LIHC_GSE140228 Smartseq2 Dataset. (D) Violin plot of the expression level of DHX34 in immune cells in LIHC_GSE146115 dataset. (E) The cell type distribution and DHX34 expression in LIHC_GSE146115 dataset.

Utilizing the scRNA-seq TISCH2 database, we procured eight distinct LIHC datasets for single-cell analysis to investigate the relationship between immune cell distribution and DHX34 expression levels at the single-cell level. Our analysis of the LIHC_GSE140228 Smartseq2 and LIHC_GSE146115 datasets revealed that monocytes or macrophages exhibited higher expression levels of DHX34 (Fig. 11A). Furthermore, we obtained insights into the distribution and expression of DHX34 across different immune cells through violin plot and clustered plots of scRNA-seq data (Fig. 11B-E). These findings suggest a significant correlation between DHX34 expression levels and the types and proportions of immune cells in LIHC.

### The immunotherapy and chemotherapy response analysis of DHX34

**Figure 12:**
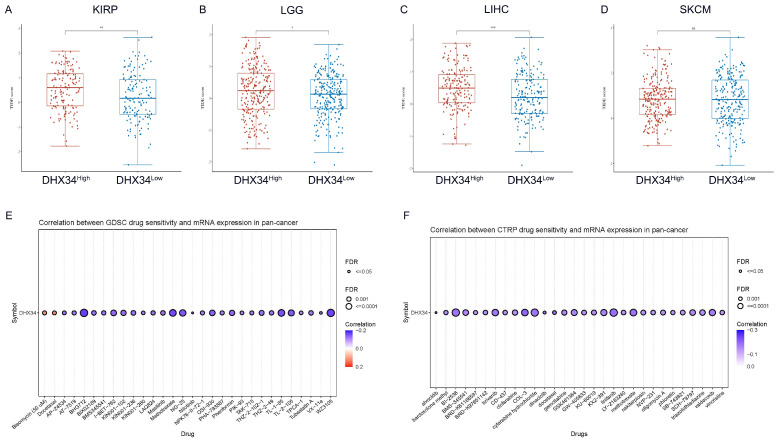
TIDE score and drug sensitivity based on DHX34 expression. (A-D) TIDE scores between the DHX34-high and DHX34-low groups in KIRP, LGG, LIHC, SKCM. (E-F) The predictive value of DHX34 for GDSC and CTRP drugs therapy in pan-cancer. (* *p* < 0.05; ** p < 0.01; *** *p* < 0.001).

To evaluate the clinical potential of DHX34 in immunotherapy, we analyzed the ICI responses in DHX34 high and low samples across various cancer types. Employing the Tumor Immune Dysfunction and Exclusion (TIDE) algorithm, we estimated the potential ICI response. Our calculations revealed that the DHX34 low group exhibited lower TIDE scores in KIRP, LGG, LIHC, and SKCM, indicating that lower DHX34 expression predicts a more favorable ICI treatment response in these cancers (Fig. 12A-D).

For drug therapy, DHX34 was found to be inversely correlated with the sensitivities of most drugs. Notably, BHG712, WZ3105, and Methotrexate emerged as the top three drugs with the highest negative correlation in the GDSC database (Fig. 12E). Similarly, COL-3, docetaxel, and linifanib ranked as the top three drugs with the strongest negative correlation in the CTRP database (Fig. 12F). These findings suggest that DHX34 may serve as a potential biomarker for predicting drug therapy responses.

### Correlation of DHX34 with ferroptosis and m6A-related genes in LIHC

**Figure 13:**
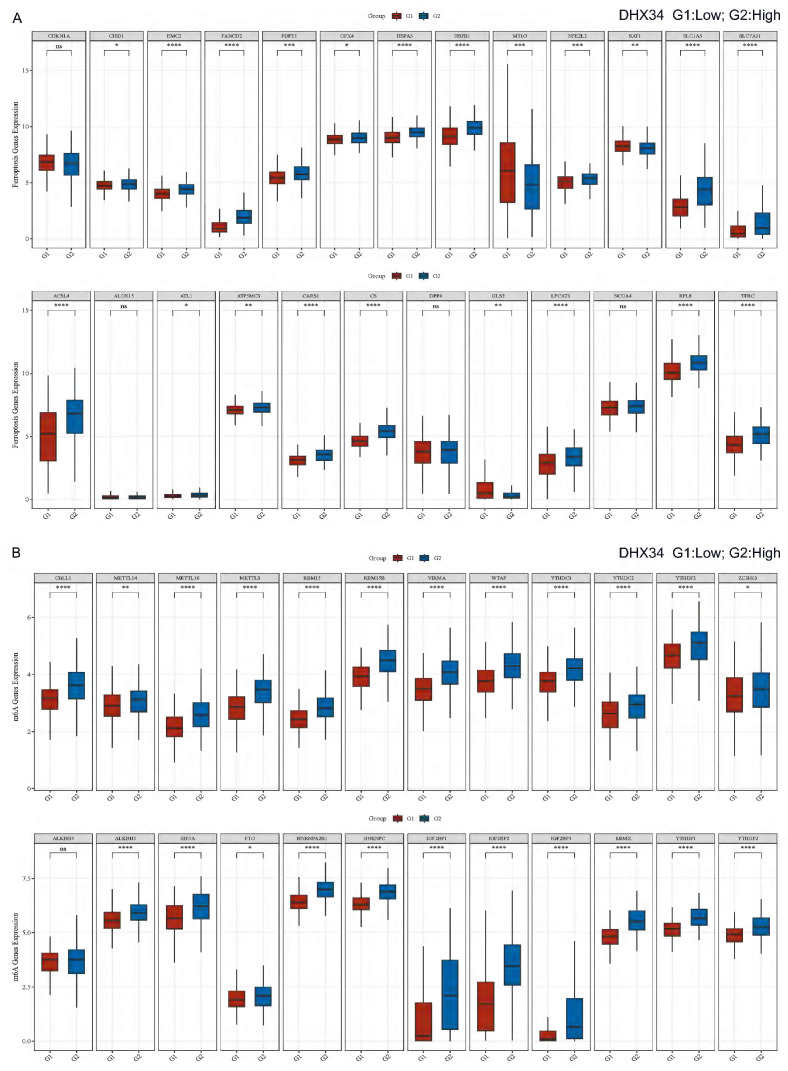
Correlation of DHX34 with Ferroptosis and m6A-related genes in LIHC. (A) Correlation of DHX34 with Ferroptosis-related genes in LIHC, (B) Correlation of DHX34 with m6A-related-related genes in LIHC.

In LIHC, we performed a correlation analysis between DHX34 expression and Ferroptosis-related genes, and found that DHX34 was significantly correlated with most Ferroptosis-related genes (CISD1, EMC2, FANCD2, FDFT1, GPX4, HSPA5, HSPB1, MT1G, NFE2L2, SAT1, SLC1A5, SLC7A11, ACSL4, ATL1, ATP5MC3, CARS1, CS, GLS2, LPCAT3, RPL8, TFRC) (Fig. 13A). We also found that DHX34 expression was significantly correlated with most m6A-related genes (CBLL1, METTL14, METTL16, METTL3, RBM15, RBM15B, VIRMA, WTAP, YTHDC1, YTHDC2, YTHDF3, ZC3H13, ALKBH5, EIF3A, FTO, HNRNPA2B1, HNRNPC, IGF2BP1, IGF2BP2, IGF2BP3, RBMX, YTHDF1, YTHDF2) (Fig. 13B).

### Experimental validation based on clinical samples

**Figure 14:**
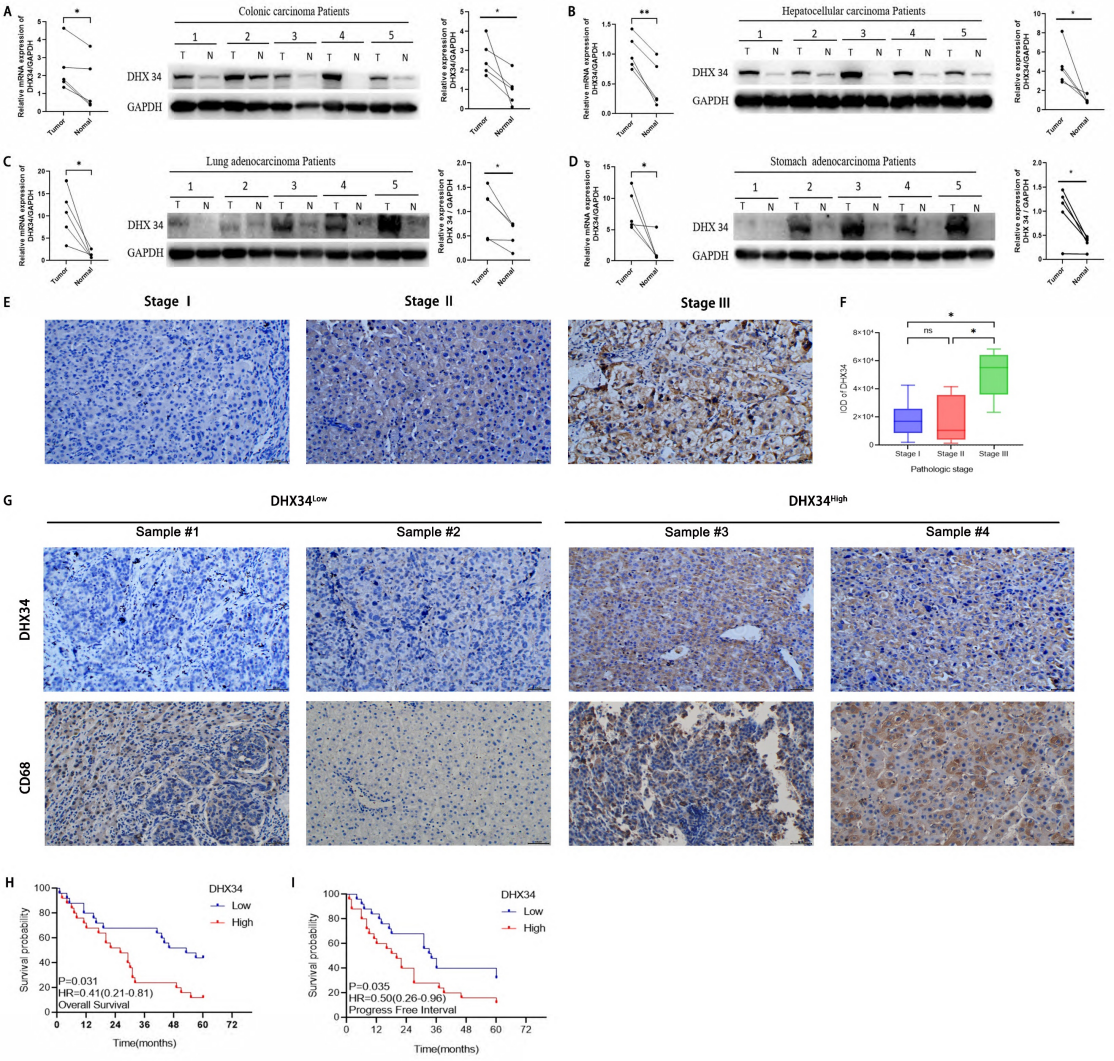
Experimental validation based on clinical samples. (A-D) Analysis of DHX34 expression in (A) colonic carcinoma, (B) LIHC, (C) LUAD, (D) STAD via qRT-PCR and WB. (E) IHC images of DHX34 expression in LIHC patients with different pathologic stages. (F) Correlation analysis of DHX34 expression and pathologic stages in LIHC. (G) IHC images of DHX34 and CD68 expression in LIHC. (H, I) Correlations of DHX34 with OS and PFI in LIHC. “T” indicates tumor, and “N” indicates normal. (ns: no significance; * *p* < 0.05).

The expression of DHX34 was further validated in our cancer cohorts using qRT-PCR and WB among 4 different types of cancer, including colonic carcinoma, LIHC, LUAD, and STAD. As shown in Fig. 14A-D, DHX34 was overexpressed in those cancer tissues compared to their corresponding non-tumor tissues. Specifically, we randomly selected 24 LIHC samples and analyzed the correlation between the expression level of DHX34 and the pathological stage. IHC results revealed a positive correlation between high DHX34 expression and advanced pathologic stages in LIHC (Fig. 14E-F). Moreover, we randomly selected 6 LIHC samples and analyzed the correlation between DHX34 expression levels and CD68 expression levels. DHX34 expression exhibited a positive correlation with CD68 expression in LIHC (Fig. 14G). Finally, we analyzed the correlation of DHX34 expression level with OS and PFI in 50 LIHC samples. Survival analysis further indicates that patients with higher DHX34 expression exhibit shorter OS (HR = 0.41, 95% CI: 0.21-0.81, p = 0.031) and PFI (HR = 0.50, 95% CI: 0.26-0.96, p = 0.035) in LIHC (Fig. 14H-I).

## Discussion

The present study focused on elucidating the function of DHX34 in pan-cancer through a bioinformatics approach. Initially, we examined the expression levels of DHX34 across various human organs and tissues. Subsequently, a comparison was made between the mRNA and protein expression levels of DHX34 in tumor tissues versus those in normal tissues. Additionally, we delved into the prognostic and diagnostic significance of DHX34 in diverse cancer forms. Furthermore, we explored the genetic variants of DHX34. Then, we detected the correlation of DHX34 expression with both TMB and MSI in pan-cancer. To further understand the functional annotation of DHX34, we constructed PPI and GSEA networks. Moreover, we examined the interplay between DHX34 expression levels and TIME in pan-cancer. Finally, we explored the relationship between DHX34 expression and the sensitivity of cancers to immune or targeted therapies. This comprehensive study provides insights into the role of DHX34 as a therapeutic target in pan-cancer.

TCGA data analysis showed that DHX34 was found in BLCA, BRCA, CHOL, COAD, ESCA, HNSC, KIRC, KIRP, LIHC, LUAD, LUSC, PRAD, READ, STAD, THCA, UCEC were highly expressed in these tumors, In the experimental validation part of this paper, we collected clinical samples from four tumors, COAD, LIHC, LUAD, and STAD, and verified the expression of DHX34 in these tumors using PCR and Western bolt, which showed that DHX34 was highly expressed in these tumors. We chose these four tumors for experimental validation for the following reasons: These four tumors are of great significance due to their high incidence and mortality rates globally; meanwhile, our institution has relatively abundant clinical sample resources for these cancer types, which facilitates the conduct of high-quality validation experiments. In addition, the high expression results of these cancer types in TCGA data are particularly significant, suggesting that DHX34 may play an important role in their development, which is worthy of further clinical validation; Considering the depth and breadth of the study and the limited resources, it is reasonable to selectively focus on those cancer types with the most significant high expression trend and prognostic value; Although the current study focused on four cancer types, COAD, LIHC, LUAD, and STAD, we also recognize that DHX34 may be equally important in other tumor types. Future studies will consider expanding the validation scope to include BRCA, CHOL PRAD, etc., to comprehensively assess the expression pattern and potential function of DHX34 in a wide range of tumors.

Alterations in the DHX34 gene were observed in approximately 5% of pan-cancer patients, with amplification representing the largest proportion of these changes. Additionally, an analysis of mutation frequencies of the DHX34 gene indicated that amplification emerges as the most prevalent type. Consistent with these findings, the analysis of data from TCGA and GEO databases revealed that the expression of DHX34 is significantly elevated in the majority of malignancies when compared to normal tissues. Given that the AUC of the ROC exceeded 0.7 in 17 malignancies and 0.9 in 7 cancers, increased DHX34 expression holds promise as a novel diagnostic marker in clinical practice. To further assess the predictive significance of DHX34 in malignancies, we employed survival analysis and found that high DHX34 expression in ACC, LGG, MESO, and SARC is associated with poor OS. Similarly, high expression of DHX34 in ACC, LGG, HCC, MESO, and SARC is predictive of poor DSS. Additionally, high DHX34 expression in ACC, KIRP, LGG, HCC, and SKCM is indicative of poor PFI.

The correlation analysis, logistic regression analysis, and subgroup analysis revealed a significant association between DHX34 and multiple clinical-pathological factors associated with cancers. Crucially, our qRT-PCR, WB, and IHC experiments confirmed that both the mRNA and protein expression of DHX34 are elevated in tumor tissues compared to normal tissues. Especially in LIHC, a high expression of DHX34 coincides with a high expression of CD68, suggesting an enhanced macrophage infiltration. However, interestingly, the TISIDB database reveals a negative correlation between DHX34 expression and monocyte expression in LIHC. This discrepancy can be attributed to two primary factors. Firstly, our clinical sample size was limited, necessitating an expansion of the sample pool for further validation. Secondly, the diverse algorithms employed for tumor immune infiltration analysis may yield varying analytical outcomes.

TMB and MSI are dependable indicators of prognosis and immunotherapeutic impact in several tumors 34, 35. Studies have demonstrated a heightened response to immunotherapy in tumors exhibiting high levels of both TMB and MSI 36,37. Consistent with these findings, our analysis revealed a positive association between DHX34 expression and both TMB and MSI in some tumor types. We, therefore, hypothesize that cancer patients with high DHX34 expression would experience improved survival following immunotherapy. This result underscores the potential of DHX34 as a novel therapeutic target for immunotherapy in cancer treatment.

To gain more insight into the biological role of DHX34, a PPI network was constructed. This analysis identified ten hub genes, and we subsequently explored their relationship with DHX34 expression across various cancer types. These hub genes exhibited a positive correlation with DHX34 expression, suggesting their comparable involvement in cancer biology. Among these hub genes, Cell Division Cycle 40 Homolog (CDC40) plays a pivotal role in enhancing cell cycle progression, cell proliferation, and migration in LIHC 38. Cell Division Cycle 5-Like (CDC5L), a regulator of the G2/M transition in the cell cycle, has demonstrated potential oncogenic activity in colorectal tumors, bladder cancer, cervical tumors, and osteosarcoma 39-42. Furthermore, upregulated Pre-mRNA Processing Factor 19 (PRPF19) expression is associated with poorer outcomes in tongue cancer patients 43. UPF1 modulates TOP2A activity and maintains stemness in colorectal cancer, thereby increasing chemoresistance to oxaliplatin 44. Additionally, UPF3a may contribute to the aggressive nature and unfavorable prognosis of colorectal cancer 45. More importantly, utilizing the GSCALite tool, we discovered that DHX34 and their ten hub genes may promote LIHC progression through the regulation of the cell cycle.

Our GSEA results further revealed that DHX34 positively correlates with the processes of cell cycle and mitosis, encompassing chromosome organization and sister chromatid segregation. Previous studies have demonstrated that abnormally expressed cancer-related genes can foster cancer development by accelerating the cell cycle. For instance, ERCC6L enhances the malignancy of breast cancer and promotes the development of mammary neoplasia by speeding up the cell cycle 46. Similarly, in gastric cancer, HER2 fuels tumor growth by regulating cell mitotic progression through the Shc1-SHCBP1-PLK1-MISP pathway 47. Our findings suggest that the high expression of DHX34 genes in tumor cells may promote mitosis and expedite the cell cycle, thereby contributing to accelerated tumor growth. Furthermore, DHX34 positively correlated with the process of gene transcription regulation, which involves sequence-specific DNA binding, transcription regulator activity, and DNA-binding transcription factor activity, which aligns with previous research on DHX9. It was shown that DHX9 supports NF-κB-mediated transcriptional activity by increasing p65 phosphorylation and nuclear translocation, another, DHX9 interacts with p65 and RNA polymerase II to bolster the expression of NF-κB's downstream targets, such as Snail and Survivin, thus intensifying the cancerous characteristics of colorectal cancer 48. Another study revealed that HIF1A-As2 epigenetically activates MYC by attracting DHX9 to the MYC promoter, thereby promoting the transcription of MYC and its target genes in KRAS-driven non-small cell lung cancer 49. Our findings collectively suggest that DHX34, through its involvement in cell cycle regulation and gene transcription, may play a pivotal role in tumor development and progression.

The TIME plays a crucial role in tumor progression and immunotherapy, as evidenced by an increasing number of studies 50,51. Utilizing the TISIDB database, we observed a negative correlation between the expression of DHX34 and T cells. T cells, which occupy a pivotal position in the immune system, are responsible for recognizing and eliminating tumor cells. However, as the expression of DHX34 intensifies, it appears to suppress the function or quantity of T cells. Consequently, this suppression enables tumor cells to evade immune attack, ultimately contributing to tumor growth and dissemination. Additionally, our analysis of the scRNA-seq TISCH2 database revealed that the expression level of the DHX34 gene is highest in monocytes or macrophages in LIHC. we, therefore, hypothesize that DHX34 may enhance the function or quantity of tumor-associated macrophages, further exacerbating the growth and aggressiveness of the tumor.

Cancer patients exhibiting elevated TIDE scores are predisposed to tumor immune escape, leading to a decreased response rate to immunotherapy with ICI 52,53. Notably, a correlation was observed between DHX34 expression and TIDE score in KIRP, LGG, LIHC, and SKCM, suggesting that DHX34 could serve as a predictor for ICI therapy responsiveness. Furthermore, our research has uncovered an association between DHX34 overexpression and reduced sensitivity of cancer cells to multiple anticancer drugs, which provides a compelling rationale that DHX34 may act as a target for cancer-specific chemotherapeutic agents.

While DHX34's impact on pan-cancer was discussed, it is important to acknowledge that we did not look into the molecular mechanism of DHX34 in malignancies in our study. In the future, more research on the mechanism of DHX34 in malignancies will be required. In summary, our investigation clarified the function of DHX34 in pan-cancer from several perspectives, including its relationship to mutational status, TMB, MSI, diagnosis, prognosis, clinical features, PPI, GESA, TIME, TIDE, and drug sensitivity, suggesting that it may be a viable diagnostic and prognostic marker for a variety of malignancies.

## Figures and Tables

**Table 1:** Correlation of DHX34 expression level with clinicopathological features in TCGA-LIHC

Characteristics	Total (N)	OR (95% CI)	P value
Gender (Male vs. Female)	374	0.627 (0.405 - 0.971)	**0.036**
Age (> 60 vs. <= 60)	373	0.657 (0.436 - 0.988)	**0.044**
BMI (> 25 vs. <= 25)	337	0.744 (0.485 - 1.143)	0.177
Pathologic T stage (T3&T4 vs. T1&T2)	371	1.268 (0.791 - 2.031)	0.324
Pathologic N stage (N1 vs. N0)	258	2.644 (0.271 - 25.763)	0.402
Pathologic M stage (M1 vs. M0)	272	0.901 (0.125 - 6.488)	0.917
Pathologic stage (Stage III & Stage IV vs. Stage I & Stage II)	350	1.391 (0.858 - 2.254)	0.180
Tumor status (With tumor vs. Tumor free)	355	1.456 (0.954 - 2.220)	0.081
Residual tumor (R1&R2 vs. R0)	345	1.019 (0.394 - 2.631)	0.970
AFP (ng/ml) (> 400 vs. <= 400)	280	5.773 (2.967 - 11.233)	**< 0.001**
Albumin (g/dl) (>= 3.5 vs. < 3.5)	300	0.998 (0.582 - 1.711)	0.993
Prothrombin time (> 4 vs. <= 4)	297	0.572 (0.345 - 0.950)	**0.031**
Child-Pugh grade (B&C vs. A)	241	0.809 (0.332 - 1.971)	0.641
Fibrosis ishak score (5&6 vs. 0&1/2&3/4)	215	0.920 (0.526 - 1.606)	0.768
Histologic grade (G3&G4 vs. G1&G2)	369	3.092 (1.982 - 4.822)	**< 0.001**
Vascular invasion (Yes vs. No)	318	1.539 (0.967 - 2.450)	0.069
Adjacent hepatic tissue inflammation (Mild & Severe vs. None)	237	1.339 (0.802 - 2.237)	0.265
